# Millimeter-scale niche differentiation of N-cycling microorganisms across the soil-water interface has implications for N_2_O emissions from wetlands

**DOI:** 10.1093/ismejo/wraf062

**Published:** 2025-05-03

**Authors:** Yu-Jia Cai, Hong-Yang Zhang, Xiao-Ran Hu, Yu-Chen Yang, Christina Hazard, Graeme W Nicol, Ji-Zheng He, Ju-Pei Shen, Zi-Yang He, Lu Zhang, Jing-Hui Zhang, Hao Liu, Sha Zhang, Zheng Chen

**Affiliations:** Department of Health and Environmental Sciences, Xi’an Jiaotong-Liverpool University, 111 Ren’ai Road, Suzhou, Jiangsu Province 215123, China; Department of Geography and Planning, School of Environmental Sciences, University of Liverpool, Brownlow Hill, Liverpool L697ZX, United Kingdom; Department of Health and Environmental Sciences, Xi’an Jiaotong-Liverpool University, 111 Ren’ai Road, Suzhou, Jiangsu Province 215123, China; Department of Health and Environmental Sciences, Xi’an Jiaotong-Liverpool University, 111 Ren’ai Road, Suzhou, Jiangsu Province 215123, China; Department of Health and Environmental Sciences, Xi’an Jiaotong-Liverpool University, 111 Ren’ai Road, Suzhou, Jiangsu Province 215123, China; Université Claude Bernard Lyon 1, CNRS, INRAE, VetAgro Sup, Laboratoire d'Ecologie Microbienne, Villeurbanne 69622, France; Université Claude Bernard Lyon 1, CNRS, INRAE, VetAgro Sup, Laboratoire d'Ecologie Microbienne, Villeurbanne 69622, France; Key Laboratory of Humid Subtropical Eco-geographical Process of Ministry of Education, School of Geographical Sciences/School of Carbon Neutrality Future Technology, Fujian Normal University, Fuzhou, Fujian Province 350007, China; School of Agriculture, Food and Ecosystem Sciences, Faculty of Science, The University of Melbourne, Parkville, VIC 3010, Australia; Key Laboratory of Humid Subtropical Eco-geographical Process of Ministry of Education, School of Geographical Sciences/School of Carbon Neutrality Future Technology, Fujian Normal University, Fuzhou, Fujian Province 350007, China; School of Agriculture, Food and Ecosystem Sciences, Faculty of Science, The University of Melbourne, Parkville, VIC 3010, Australia; Department of Health and Environmental Sciences, Xi’an Jiaotong-Liverpool University, 111 Ren’ai Road, Suzhou, Jiangsu Province 215123, China; Department of Health and Environmental Sciences, Xi’an Jiaotong-Liverpool University, 111 Ren’ai Road, Suzhou, Jiangsu Province 215123, China; Department of Health and Environmental Sciences, Xi’an Jiaotong-Liverpool University, 111 Ren’ai Road, Suzhou, Jiangsu Province 215123, China; Department of Health and Environmental Sciences, Xi’an Jiaotong-Liverpool University, 111 Ren’ai Road, Suzhou, Jiangsu Province 215123, China; Department of Health and Environmental Sciences, Xi’an Jiaotong-Liverpool University, 111 Ren’ai Road, Suzhou, Jiangsu Province 215123, China

**Keywords:** soil-water interface, Millimeter-scale, N_2_o emissions, wetlands, nitrogen cycling, niche partitioning, ammonia oxidizing microorganisms

## Abstract

Wetlands can be a significant source of N_2_O under current global climate change regime with the soil-water interface representing a biogeochemical hotspot for microbial activity. However, the role of soil-water interface in controlling N_2_O emissions remains poorly understood. We hypothesized that the millimeter-scale redox gradient across the soil-water interface generates corresponding distinct niche for N-cycling microorganisms that collectively regulate the production and consumption of N_2_O over the same spatial scale. The abundance, transcriptional activity and spatial organization of different N-cycling guilds across the soil-water interface were characterized in mesocosms from three different paddy soils with different N_2_O emissions. Results demonstrated millimeter-scale stratification of N-cycling microbial activity across the soil-water interface, and in particular within the first 10 mm of flooded soils. Ammonia-oxidizing microorganisms were only transcriptionally active in the top 4 mm, suggesting a previously underestimated contribution to N_2_O emissions from wetlands. Variation in N_2_O accumulation was observed across the soil-water interface, with the highest concentrations measured at either the soil-water interface or in the deeper anoxic layer of paddy soils. Despite this difference, N_2_O-reducing microorganisms exhibited high transcriptional activity at the soil-water interface in all soils, suggesting that there is a microbial-mediated sink for N_2_O across the soil-water interface that can reduce N_2_O produced from both oxic and anoxic layers. This work demonstrate an underappreciated and essential role of the microbial hot zones at soil-water interface in regulating N_2_O emissions from wetlands.

## Introduction

Microbial communities contribute to the regulation the greenhouse gas (GHG) emissions from wetland soils [1–3]. Although wetlands cover only 8% of the Earth’s terrestrial surface, they represent one of the largest organic carbon stocks [3, 4]. Warming and hydrological fluctuation associated with a changing climate may enhance microbial degradation of organic carbon, and increase GHG emissions from wetlands [3, 5–8]. Among the GHGs emission from wetlands, N_2_O, a potent GHG and a depletor of stratospheric ozone layer, has received less attention compared to methane and CO_2_. However, fluxes of N_2_O from wetlands, such as paddy fields, riparian and intertidal zones, are highly variable in both space and time due to the complexity of N-cycling processes and N_2_O dynamics [3, 9–11]. Production of N_2_O occurs through microbial-driven pathways during both nitrification- and denitrification-related processes [12, 13]. The extent that these different pathways contribute to N_2_O production largely depends on the microbial community and environmental conditions. For example, nitrification-related N_2_O dominates in drier and aerated upland soils [12, 14] whereas denitrification-derived N_2_O production dominates in soils with high or saturated water content [15–18].

Recent studies have suggested that nitrification, particularly the role of Ammonia-oxidizing microorganisms (AOM) in wetlands, have been underappreciated [3, 19, 20]. Aerobic chemolithoautotrophic AOM consist of ammonia-oxidizing archaea (AOA), ammonia-oxidizing bacteria (AOB), and the more recently discovered complete ammonia oxidizers (comammox *Nitrospira*) [21, 22]. AOM can thrive at the thin oxidized layer of surface wetland soils or in the rhizosphere of vascular plants [23–25]. During ammonia oxidation N_2_O is produced via both biotic and abiotic mechanisms [26–28]. AOB can also produce N_2_O through the denitrification pathway [12, 20]. N_2_O that is produced can be directly released to the atmosphere as AOM reside in surface soils. Other N_2_O producing pathways in wetlands include incomplete denitrification and chemodenitrification [12, 29, 30], adding to the intricate and diverse means of N_2_O production.

In wetlands, water flooding reduces the aerobic layer in soil and generates a soil-water interface (SWI) that bridges the oxic overlaying water with the anoxic soil compartment. Due to the slow oxygen diffusion from the atmosphere to water and high oxygen consumption by microorganisms, a distinct oxygen gradient forms across the SWI [31–34]. When oxygen is depleted in soils microorganisms use alternative electron acceptors such as nitrate (NO_3_^−^), iron, manganese, and sulfate during respiration [35]. Such oxygen gradient can lead to the niche differentiation of nitrifiers and denitrifiers. Denitrification therefore occurs in deeper anoxic layer, with aerobic nitrification favored at the SWI where oxygen is relatively abundant [25]. Two zones for N_2_O production may exist in flooded soils, consequently creating a niche for N_2_O-consuming microorganisms across the SWI.

Oxygen and other biogeochemical gradients across the SWI generally manifest at micro- or millimeter scales [31, 32, 34, 36]. Heterogeneity in microbial communities at these scales may also be present but this is often obscured by typical bulk sampling approaches [37–39]. Considering the size of prokaryotes and the scale at which cellular interactions can occur, millimeter-scale is a biologically relevant and feasible scale to study the activities of microbial communities across the SWI [37, 38]. Although microbiome analyses have been successfully performed at this scale in soil crust [40], microbial mats [41, 42], and sediment [43], less has be done across the SWI [38].

This study aimed to examine the potential partitioning of N_2_O production pathways across the SWI to gain insight into N cycling and N_2_O dynamics in wetlands. It was hypothesized that the redox gradient across the SWI generates different niches for N-cycling microorganisms that influences the production and consumption of N_2_O across the SWI. A flooded mesocosm experiment was performed to profile the vertical distribution of N-cycling microorganisms across the SWI at the millimeter-scale. Utilizing our previously developed approach to effectively sample at the millimeter-scale [38], we assessed microbial community structure, abundance of N-cycling microorganisms, and transcriptional activity vertically at 2 mm spatial resolution. With incorporation of *in-situ* and fine-scale sampling methods for soils and sediments [44–46], spatial and temporal heterogeneity in soil water, gas, and solid phase were determined, which enabled distinguishing N-cycling processes occurring across the SWI.

## Materials and methods

### Soil collection and characterization

Wetland soils including paddy soils were collected from ten sites across China in 2017 (Table S1). The upper 20 cm layer of soil was collected. Soil samples were air-dried, sieved (2 mm) for homogenization, and preserved at room temperature. A subsample of each soil was used for soil physicochemical analyses (Table S1). Soil pH was measured in a soil suspension at a soil:water ratio of 1:2.5. Soil NH_4_^+^, NO_2_^−^, and NO_3_^−^ -N were extracted with 0.5 M K_2_SO_4_ and determined using colorimetric assays with a microplate reader (Tecan, Austria) [47].

A preliminary soil microcosm was carried out to examine the N_2_O emissions of the ten wetland soils. Three paddy soils, Kunshan (KS), Shaoguan (SG), and Wenshan (WS) (Table S1), were selected for the subsequent soil mesocosms experiment. Due to the quantity of soil required for these experiments, KS and WS paddy soils were re-collected according to the original GPS information in February 2023 and May 2023, respectively. Soil physicochemical analyses were measured as previously described (Table S2).

### Soil microcosm experiment to determine N_2_O emissions from wetland soils

Soil microcosms were established in triplicate under flooded conditions to determine N_2_O emissions from the ten wetland soils. Briefly, each 40 mL glass vial was filled with 10 g dry soil and ultrapure water to form a 3 cm water layer and an 18 mL headspace air. Microcosms were sealed with breathable sealing film and incubated in the dark at 25°C for 45 days. 1 mL headspace air was sampled for N_2_O analysis every week.

### Soil mesocosm experiment with selected soils

Of the ten soils tested, two demonstrated high rates of N_2_O emissions (KS and WS) and were used in a subsequent mesocosm experiment with a non N_2_O-emitting soil (SG) to examine N_2_O emissions across the SWI (Table S1). Soil was added into lightproof pot containers (12 cm length × 12 cm width × 15 cm height) to form a 10 cm soil layer (approximately 1300 g). Deionized water was added to the soil until saturation with a 3 cm layer of standing water. Three replicate pots were established for WS and SG soil, and four replicate pots for KS soil. Mesocosms were placed in an automated platform (Fig. S1) and covered with aluminum foil to exclude light during incubation. Room temperature was maintained at 25°C for the duration of the experiment. Water levels were checked twice a week and adjusted if needed to maintain the standing water layer. The redox potential (Eh) gradient across the SWI was determined every week using an Eh microelectrode. For KS soil, when the redox gradient across the SWI reached 400 mV after 38 days of incubation, the four mesocosms were randomly divided into two groups for light–dark cycling to imitate the natural environment. One group, designated as “light–dark”, was subjected to a 12 h light- 12 h dark cycle, whereas the other group “all-dark” was kept in constant darkness. The light–dark cycling was maintained for 6 days to prevent the formation of phototrophic microbial biofilm. At the end of the incubation (44 days for KS, 40 days for SG and WS soils), soil cores within each mesocosm were destructively sampled for analyses of soil chemistry (e.g., NH_4_^+^ and NO_3_^−^), microbial community structure, and transcriptional activity.

### Determination of N concentrations and N_2_O fluxes across SWI

Soil NH_4_^+^, NO_2_^−^, and NO_3_^−^ -N concentrations were determined in either the porewater or soil extracts with soil porewater collected using the integrated porewater injection (IPI) sampler [44, 46] (Detailed in Supplementary text). The IPI sampler, allowing in situ and continuous sampling, was used in the KS soil to monitor the change of dissolved NH_4_^+^, NO_2_^−^, and NO_3_^−^ -N across the SWI during incubation. Whereas for SG and WS soils, soil cores were sampled for N analysis at the end of incubation. Briefly, soil cores were sampled using a 20 mL modified syringe, carefully inserted into the soil to prevent disturbance of the SWI before withdrawing to remove a core [38]. After sampling, the soil core was pushed out at 2 mm intervals and slices removed using a sterile ceramic knife. The 2 mm soil slice were put to inorganic N extraction at a soil: 1 M KCl ratio of 1:5.

Soil gases (e.g., N_2_O and CO_2_) were analyzed after diffusing into silicone tubing (5 mm outer diameter × 115 mm length). The silicone tubing was attached to the inside of the pot using silicone sealant while isolating the water [48–50]. A gas tight syringe was inserted into the silicone tubing and 1 mL gas was collected. The spatial resolution for the soil N_2_O profile was 10 mm.

To determine N_2_O fluxes in the mesocosms, gas was collected once or twice a week using the static chamber technique with transparent polymethylmetacrylate (PMMA) chambers (10 cm length × 10 cm width × 6 cm height). During sampling, the overlaying water within each mesocosm seals the chamber (Fig. S1C). Preliminary tests showed that a 2 h interval was appropriate for sealing chambers to measure N_2_O emissions (data not shown). During the 12 h light-12 h dark cycling, the N_2_O flux measurement was performed 6 h into the lighting period of incubation every day.

The N_2_O flux was calculated using linear regression analysis [51, 52]:


$$ F=\left(\frac{\Delta c}{\Delta t}\right)\times \frac{V}{22.4}\times \left(\frac{273.15}{273.15+T}\right)\times \frac{M}{A} $$

Where F is the N_2_O flux (ng N h^−1^ dm^−2^), Δc/Δt is the rate of N_2_O accumulation in the chamber (nmol mol^−1^ h^−1^), V is the effective sampling volume of the chamber (L), 22.4 is the molar gas constant at 0°C (L mol^−1^), T is the temperature in Celsius (°C), M is the molecular weight of N_2_O-N (g mol^−1^), and A is the surface area of the chamber (dm^2^).

For N_2_O analysis, 1 mL of gas sample was injected into a GC fitted with an ECD (Agilent 7890B, USA). Carboxen 1010 columns were kept at 150°C (oven temperature), and ultrapure N_2_ was used as carrier gas. The ECD current was 250 mV, and the ECD cell was kept at 300°C.

### N-cycling microbial community structure and transcriptional activity across the SWI

Approximately 1.0 g per soil slice (2 mm) was used for immediate RNA extraction and another 0.4 g was stored −20°C for subsequent DNA extraction. Total RNA was extracted using the PowerSoil Total RNA Isolation Kit (Qiagen, USA) according to the manufacturer’s instructions. Removal of genomic DNA and reverse transcription were performed with the PrimeScript RT reagent kit with gDNA Eraser (Takara, Japan). DNA extraction was performed using the DNeasy PowerSoil Kit (Qiagen, USA), following the manufacturer’s instructions. Nucleic acid concentration and purity were determined using a NanoDrop One spectrophotometer (Thermo Scientific, USA).

Quantitative PCR (qPCR) was conducted in a Light Cycler 480 II (Roche, Switzerland) with three technical replicates per sample. Primer sets used for qPCR of N-cycling and 16S rRNA genes are given in Table S3. For the nitrite-oxidizing bacteria (NOB), the primer set nxrB169f/nxrB638r was designed based on *Nitrospira nxrB* gene and unlikely detect other NOB here due to the dominance of *Nitrospira* NOB in low-NO_2_^−^ and low-oxygen environments (e.g., wetland) [53, 54]. The reaction mixture (10 μl per sample) consisted of 5 μl TB Green premix (Takara, Japan), 0.5 μl for forward and reverse primer (final concentration 0.5 μM), 1.5 μl total cDNA/DNA, and 2.5 μl DNase-free water. Standard curves for quantification were generated using 10-fold serial dilutions of plasmid DNA containing a fragment of each target gene. Amplification efficiency of standards ranged from 95%–110% with an *R*^2^ > 0.99. Melt curve analysis and gel electrophoresis were conducted to assess the specificity of qPCR products.

### Statistical analysis

Pearson correlation analysis was performed for the abundance of N cycling microorganisms using the R packages “Hmisc” [55] and “corrplot” [56]. ANOVA and t-test were performed using IBM SPSS Statistics (Version 29.0.0.0).

## Results

### N_2_O emission and depth profiles vary in wetlands

The emissions of N_2_O from the wetland soil samples, collected from rice paddies, coastal intertidal wetland, and other wetlands across China, were measured after 45 days of incubation under water-flooding conditions (Table S1). Two soils that produced detectable N_2_O emissions (KS and WS) and one soil (SG) that did not were selected for the subsequent mesocosm experiments.

**Figure 1:**
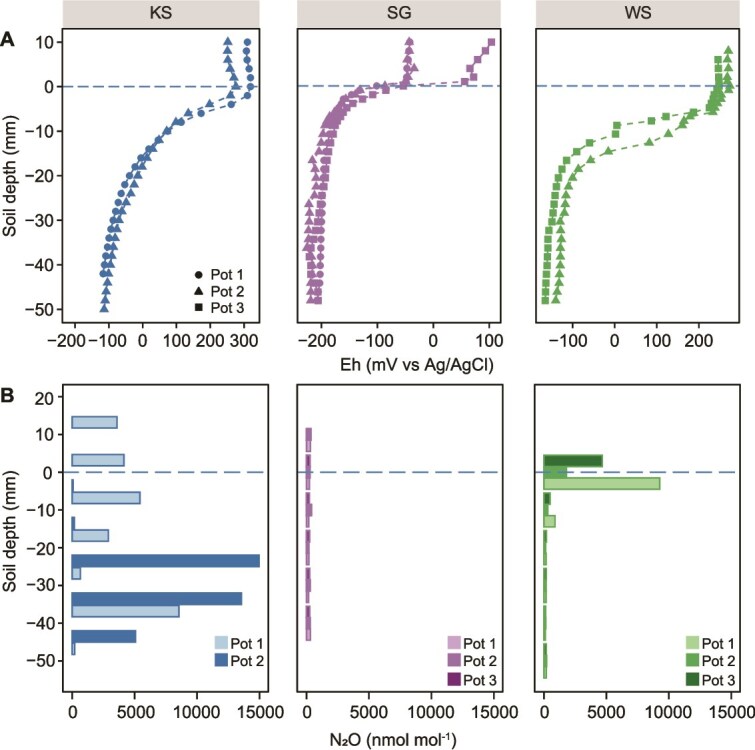
Depth profiles of eh (A) and N_2_O (B) across the soil-water interface at the end of incubation experiment. Additional graphs showing the eh gradient change during incubation of KS soil can be found in Supplementary Fig. S4A. Different symbols in (A) and shades of the color in (B) represent independent replicates.

Various parameters (Eh, N_2_O, NH_4_^+^, NO_2_^−^, and NO_3_^−^) were measured across the SWI (from the SWI to the 50 mm soil depth) at the end of the incubation experiment (Fig. 1, S2, and S3). The Eh dropped at ~300 mV from the water layer to the ~10 mm soil layer in all three soils (Fig. 1A), representing a steep redox gradient across the SWI at the millimeter scale. Based on this redox gradient, the soil was divided into 3 layers: an oxic layer (0–2 mm), a transition layer (2–10 mm), and an anoxic layer (below 10 mm).

**Figure 2:**
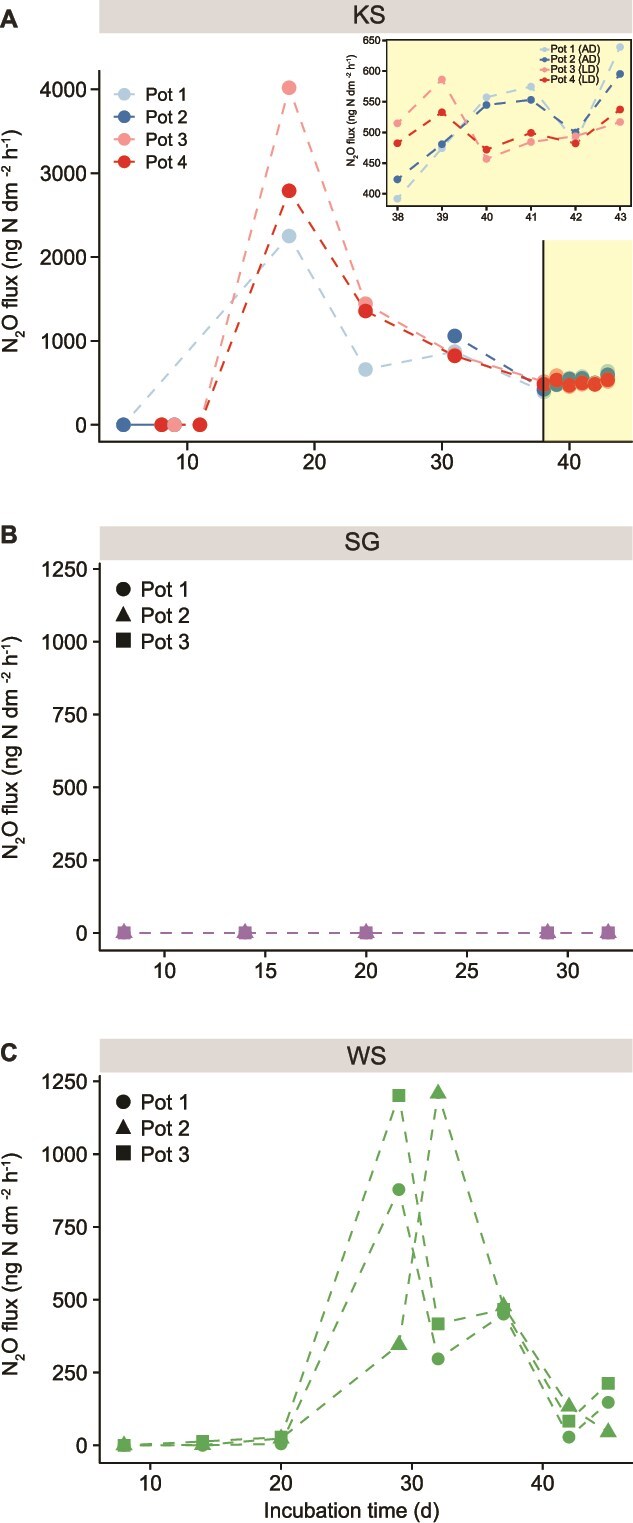
N_2_O flux of the (A) Kunshan (KS), (B) Shaoguan (SG) and (C) Wenshan (WS) soil during the soil incubation experiment. In (A) KS soil, the four mesocosms were incubated in the dark and can be considered as replicates until day 38 before separated into the all-dark (blue) and light–dark (red) groups. The N_2_O flux during light exposure was zoomed in on the top of (A).

During the incubation, only the KS and WS soils produced N_2_O with emission rates peaking with 7.00 ± 2.10 and 1.32 ± 0.70 μmol mol^−1^ h^−1^, which then decreased to 1.11 ± 0.50 and 0.13 ± 0.08 μmol mol^−1^ h^−1^ after 40 days, respectively (Fig. 2). The depth profiles of N_2_O concentrations varied with different N_2_O accumulation zone across the SWI. N_2_O was more accumulated in the 20–40 mm soil layer of KS soil, whereas in WS soil the accumulation occurred at the SWI, and no accumulation was found in SG soil (Fig. 1B).

In the flooded soils, the concentration of NH_4_^+^ was greater than that of NO_2_^−^ and NO_3_^−^ across the depth profile (Fig. S2). Concentrations of NH_4_^+^ increased with depth in KS and WS soils (Fig. S2A). Temporal change of dissolved NH_4_^+^ profile was tracked in KS soil using the IPI sampler (detailed in Supplementary Material). The gradient of NH_4_^+^ concentrations across the SWI developed and reached its steepest slope at day 25, then decreased to 0.5–3.5 mg L^−1^ (Fig. S2A and S3), which was higher than the original NH_4_^+^ content in bulk soil.

### Abundance of nitrifier functional genes and transcripts across the SWI

**Figure 3:**
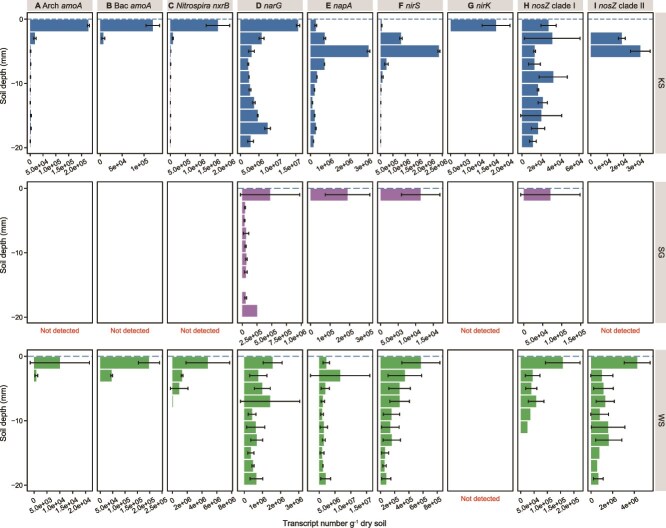
Abundance of transcripts across the soil-water interface of genes encoding for N-cycling processes. Error bar represents the standard error of the mean of technical replicates of KS soil microcosm and biological replicates (n = 3) of SG and WS mesoocosms. No biological replicates are available for KS soil. Additional graphs showing the transcript abundance of N-cycling genes in the KS 0–50 mm soil profile are presented in Supplementary Fig. S6.

**Figure 4:**
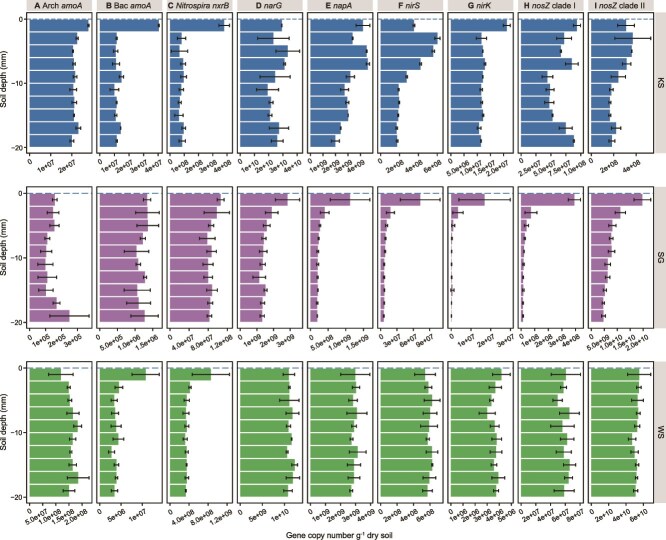
Abundance of genes across the soil-water interface encoding for N-cycling processes. Error bar represents the standard error of the mean of technical replicates of KS soil mesoocosm and biological replicates (n = 3) of SG and WS mesocosms. No biological replicates are available for KS soil. Additional graphs showing the gene abundance of N-cycling genes in the KS 0–50 mm soil profile are presented in Supplementary Fig. S6.

The quantification of nitrifier abundance and transcriptional activity indicated that nitrification primarily occurred within the top 4 mm (Fig. 3A-C and 4A-C). The AOA ammonia monooxygenase sub-unit A (*amoA*), AOB *amoA,* and *Nitrospira* nitrite oxidoreductase beta sub-unit (*nxrB*) genes and transcript abundances were highest in the top 0–2 mm surface soil (*P* < 0.001), with mRNA transcripts only detected in 0–4 mm layers in KS and WS soils, and no nitrification transcriptional activity detected in SG soil profile. No depth-related trends were observed for total prokaryotic 16S rRNA gene and transcript abundances (Fig. S4). Among the three soils, nitrifiers were most abundant in WS soils, followed by KS and SG soils (*F* = 59.2, *P* < 0.001).

### Abundance of NO_3_^−^ and NO_2_^−^ reductase genes and transcripts across the SWI

We quantified marker genes for denitrifiers, including those encoding the membrane-bound and periplasmic NO_3_^−^ reductase sub-units (*narG* and *napA*), as well as the cytochrome cd1-containing and copper-dependent NO_2_^−^ reductase sub-units (*nirS* and *nirK*). Unlike the nitrifiers, KS soil harbored more NO_3_^−^ and NO_2_^−^ reducing microorganisms followed by WS and SG soil (*F* = 190.3, *P* < 0.001). These denitrifier associated genes demonstrated different distribution patterns compared to those of nitrifiers, as well as differed within their N-cycling functional equivalent and distinct groups (Fig. 3, 4, and S5). Changes in transcript abundance of *narG* and *napA* genes demonstrated similar depth-dependent patterns in KS and WS soil profiles, with *napA* gene more transcriptionally active in the transition zone (Fig. 3D and E). No spatial changes in gene abundance were observed (Fig. 4D and E).

For NO_2_^−^ reducers, *nirK* gene and transcripts were generally lower (or absent) throughout the profile compared to *nirS* (Fig. 3F,G and 4F,G). The transcriptional activity of *nirS* and *nirK* genes varied in different soil profiles (Fig. 3F,G and S5G,H). Transcripts of *nirS* were more abundant at 2–6 mm in KS soil but decreased with increasing soil depth in WS soil (Fig. 3F and S5G). The transcripts of *nirK* guild were only detected at the SWI of KS soil, and were below detection in SG and WS soil profiles.

### N_2_O-consuming microorganisms across the SWI

The N_2_O reductase (NosZ) genes are separated into two distinct clades and are associated with different functional roles that clade I microorganisms are primarily denitrifiers, whereas clade II are non-denitrifying pure N_2_O reducers [57]. The abundance of N_2_O-reducing microorganisms varied significantly (*F* = 711.7, *P* < 0.001) in the order WS > SG > KS soil, but corresponding order was not found in their transcriptional activity with few active N_2_O reducers found in SG soil. Both *nosZ* clades were transcriptionally active throughout the SWI in KS and WS soils, and were more active closer to the SWI (Fig. 3H,I and S5I,J). No obvious depth-related changes in gene abundance were observed. The two clades exhibited divergent transcriptional patterns across the SWI. In KS soil, clade I *nosZ* transcripts were detected throughout the profile, whereas the activity of clade II was restricted to the first 6 mm. In contrast, the transcriptional activity of both clades decreased with increasing depth in WS soil, with the highest activity observed at the SWI.

### Effect of light exposure on N-cycling microorganisms at the SWI

To investigate the response of N-cycling microorganisms at the SWI to light exposure, KS soil mesocosms were incubated under a diurnal cycle for 6 days at day 38. Relatively lower N_2_O emission were observed during light exposure (Fig. 2A), concurrent with increased growth and activity of N-cycling microorganisms at the SWI (Fig. S5). Microbial responses to light exposure varied in terms of gene abundances and transcriptional levels. For example, after 6-day light–dark cycling, a congruent stimulation of both gene abundances and transcriptional levels in AOA, AOB, *napA* NO_3_^−^ reducers, NO_2_^−^ reducers, and clade I *nosZ* N_2_O reducers were observed (Fig. S5). Clade II *nosZ* N_2_O reducers were only stimulated at the transcriptional level. Light exposure had no effect on *Nitrospira nxrB* and *narG* NO_3_^−^ cohorts. A greater number of significant positive correlations were found between the abundances of N-cycling microorganisms in the light–dark group compared to the all-dark group (Fig. S6).

## Discussion

**Figure 5:**
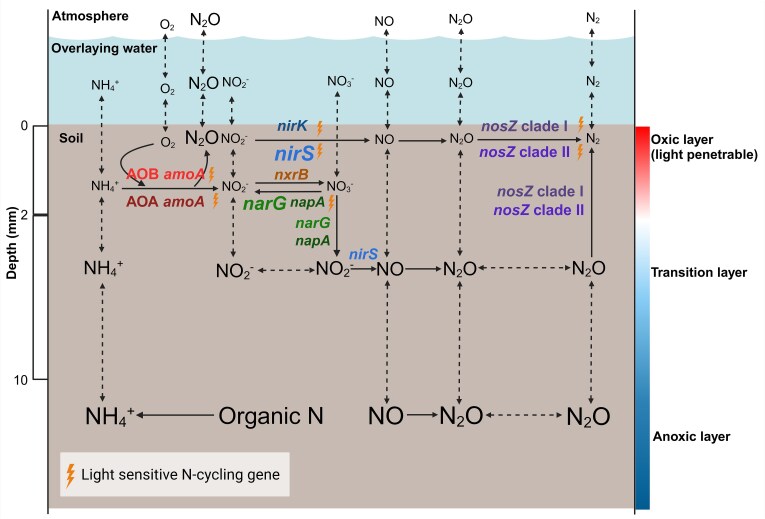
Schematic description of active N transformation processes across the soil-water interface. The size of the N species label indicates relative differences in concentration. Solid arrows indicate microbial transformations and dotted arrows indicate N diffusion or exchange. Microbial guilds possessing the same gene type are described with the size of the label indicating the relative difference of guild activity. *nxrB* represents *Nitrospira* microorganisms which thrive in low-NO_2_^−^ and low-oxygen environments [54]. Lightning symbol next to the label indicates that the microbial N cohort is sensitive to light exposure.

The millimeter-scale variation of N-cycling microbial community confirmed our hypothesis that distinct spatial distributions of active N-cycling microorganisms exist across the narrow SWI, suggesting their collective impact in regulating the N_2_O emission from wetland soils (Fig. 5). Alike, the vertical stratification of microbial communities at millimeter scale is observed in diverse ecosystems, as evidenced by studies of paddy soil [33], soil crust [40], microbial mat [41, 42], and sediment [43]. Whereas these works focused on carbon, sulfur cycling and total microbial community shifts, we emphasized on the novel insights into functional redundancy and N_2_O emission through these transcriptional stratification of N-cycling genes.

### Role of SWI in N_2_O emission from wetland

According to the currently known N_2_O producing pathways, we postulated that N_2_O would be produced in deeper anoxic zones where it is suitable for denitrification, or at the SWI where nitrifiers were predicted to thrive. In the N_2_O producing soils, N_2_O accumulated in deeper anoxic zone of KS soil and at the SWI of WS soil. Such accumulation of N_2_O was also found in biofilm [58–60] and a Danish wetland [61], where the oxygen gradient manifests at micrometers and millimeters. Correspondingly, the activity of AOM were restricted at the SWI together with low ammonium concentrations implying that there was nitrifier-related N_2_O production in this zone. Nitrifiers produce N_2_O via two pathways: through ammonia oxidation and nitrifier denitrification by AOB and NOB [12, 14, 27, 62]. Intriguingly, *nirK* NO_2_^−^ reducers were more active at the SWI, relative to the AOM. The similar spatial distribution between *nirK* transcript and the activity of nitrifying microorganisms may be attributed to the close evolutionary history of *nirK* orthologue gene among nitrifiers [63]. In metagenomics studies, the *nirK* gene has been found in AOA and NOB from marine systems [26, 64, 65]. The catalytic product of NO_2_^−^ reductase—NO is not only an essential intermediate during ammonia oxidation [66], but also a signaling molecule that enhances the mutualistic interaction between AOA and *Nitrospira* [54].

The N_2_O accumulation at the SWI of WS soil was related to active N cycling microorganisms. However, the activity of denitrifying populations at the end of the incubation was not within the N_2_O accumulation zone in KS soil. We speculate that this might be attributed to the 1) distinct spatial distribution of NO reducers compared to NO_2_^−^ reducers [62]; 2) contribution of abiotic N_2_O production such as chemodenitrification [30]; 3) incomplete denitrification during the formation of the redox gradient and peak N_2_O emissions, and 4) low activity of N_2_O-consuming microorganisms. Moreover, *nir* genes may not reflect activity of upstream or downstream processes of denitrification and therefore cannot be used as a proxy for all N oxide reductive processes [67]. During N_2_O formation, NO production is usually concomitant with N_2_O [12, 29, 58]. However, current primers cannot be used to detect and quantify *norB* genes of NO reducers from a diverse range of taxonomic groups [68]. Current findings have limitation in distinguishing the source of N_2_O from the wetlands and the contribution of different N-cycling guilds, which may be addressed in future research with the application of stable isotope techniques and omics-based approach.

Combining the observed N_2_O accumulation across the SWI, SWI showed potential in reducing N_2_O emission through the highly active N_2_O-reducing microorganisms in the top 10 mm soil layer or at the SWI. This spatial separation suggests that these microorganisms act as a microbial N_2_O sink, consuming N_2_O either diffused from deeper soil or produced by nitrifier metabolism. A similar stratification between N_2_O and N_2_O-consuming microorganisms has been found in oxic marine water, where N_2_O consumption rates are higher compared to the anoxic zone [65, 67]. In addition, these aerobic N_2_O consumers can consume more N_2_O once shifted to anoxic conditions [65, 69–71]. This rapid N_2_O consumption under oxic condition is surprising, as it is typically favored in anoxic environments, suggesting that oxygen plays a key regulatory role. Studies have demonstrated that *nosZ* gene expression is regulated by oxygen level, and N_2_O reduction characterizes a life strategy for aerobic microorganisms to survive temporary anoxia [69, 71, 72]. Oxygen tolerance in N_2_O consumers is not related to the phylogeny or the structure of nitrous oxide reductase (NOS), but depends on their ability to quickly scavenge oxygen and/or the activity of accessory proteins encoded by the *nos* cluster, which maintain low oxygen levels to prevent NOS inactivation [73]. Though aerobic N_2_O reduction is promising, it remains challenging in practical applications [74]. Further research is needed to investigate the oxidative damage to denitrification reductases using detailed proteomic analyses [71]. Overall, the balance between nitrifiers and N_2_O-consuming microorganisms at the SWI plays a critical role in controlling N_2_O emission from flooded wetland soils, highlighting the underappreciated importance of the SWI.

### Niche differentiation of the N-cycling microorganisms across the SWI

N-cycling microbial activity is stratified at the millimeter-scale across the SWI, with most activity restricted within the top 10 mm of the flooded soils, and particularly at the SWI (Fig. 4). These findings are observed in biofilm [60] and microbial mat [75], yet rarely found in analogous environments due to the trade-off between fine-scale soil/sediment sampling and active microbiome analysis. The spatial variations reflect the physiological features of different N-cycling guilds. For example, the ammonia oxidizers, which require oxygen for energy conservation, thrived at the oxygen-rich SWI, whereas denitrifiers preferred the transition layer in the flooded soils.

Niche differentiation exists in functional equivalent denitrifiers. For example, the *narG* guild predominated in NO_3_^−^ reducers, which is also observed in estuarine sediment or flooded paddy soils [76, 77]. The prevailing transcriptional activity through the soil profile confirms NO_3_^−^ respiration one of the ancient energy-conserving pathway in microorganisms, and the NO_3_^−^ reductase involved are biochemically distinct [78, 79]. The membrane-bound NO_3_^−^ reductase can provide energy through the proton motive force during NO_3_^−^ reduction, whereas periplasmic NO_3_^−^ reductase does not [80]. High NO_3_^−^ concentrations seem favorable for the *narG* microbial cohorts, whereas populations possessing *napA* prefer low NO_3_^−^ concentrations and oxic conditions [79, 81, 82].

The *nirS* guild was observed as the dominant NO_2_^−^ reducing microorganisms in the flooded soils. Genomic analysis and experimental results suggest that the *nirS* guild dominates over the *nirK* guild under conditions that are favorable for denitrification [83–85]. Similarly, their transcriptional activity was dominant across the SWI whereas *nirK* transcripts were only detected at the SWI. Niche partitioning between *nirS* and *nirK* NO_2_^−^ reducers may be influenced by their tolerance to oxygen with *nirS* more sensitive to oxygen than the *nirK* carriers [83, 86–89].

### Influence of day-time light on activity of N-cycling microorganisms at the SWI

Light exposure affected the abundance and transcriptional activity of N-cycling microorganisms and slightly decreased N_2_O emissions, but did not influence N_2_O accumulation in the flooded soils. More significant correlations were found in the light–dark group, suggesting enhanced microbial interactions within the N-cycling microbial community under light. Studies have revealed the difference in N_2_O emissions under light exposure, which may be related to the diurnal change in microbial activity, and confirmed it a prevalent phenomenon across terrestrial ecosystems, including agricultural, forest, and wetland soils [90–92]. The underlain mechanisms remain less studied, but recent research from riverine water columns reported strong inhibition of N_2_O emissions with increasing light irradiation, potentially attributed to the change in N-cycling microbial activity [93]. However, the robustness of the stimulation effect on N-cycling microorganisms here requires further experimental confirmation with more replicates. The biological replicates are limited in KS soil as there was a trade-off between requirements of RNA extraction and massive sample number when investigating the SWI at fine-scale.

These light-driven changes in microbial community structure and activity could be associated with the formation of photochemically produced reactive intermediates (e.g., singlet oxygen (^1^O_2_), peroxide hydrogen (H_2_O_2_), and hydroxyl radicals (·OH)) [93]. The oxygen gradient and redox oscillation makes the SWI a previously unappreciated hotspot for production of reactive oxygen species, influencing microbial activity and N-cycling [94, 95]. Though reactive oxygen species can inhibit or kill cells, light exposure in this study stimulated the N-cycling microbial activity and interaction, indicating microbial response to oxygen stress is dose- and exposure-time dependent [96]. Metatranscriptomics may help to examine whether microorganisms at the SWI are coping with oxidative stress in the daytime.

## Conclusion

This study provides a detailed perspective on the dynamic processes of nitrification and denitrification across the SWI at the millimeter scale. Sampling at an appropriate spatial scale may help clarify the ecological processes driven by microbial activities and lead to more reliable inferences of the functional capability of microbiomes [37, 97, 98]. Findings demonstrate that the SWI is an ideal environment to disentangle the complexities of N_2_O emissions from wetlands. The SWI may harbor an underappreciated “microbial N_2_O barrier”, where the balance between nitrification-related N_2_O production and N_2_O consumption collectively controls the release of N_2_O to the atmosphere. The SWI in wetland systems characterizes as a fine-scale, sensitive, and representative oxic-anoxic environment in where soils are subjected to intermittent or permanent water flooding. Drainage, warming, and land use change of wetland soils disturb the SWI, which may cause negative effects on wetland ecosystem services through accelerating microbial activity and consequently promoting GHG production or release from deeper preserved soils.

## Supplementary Material

202503Revised_Figure_S1_wraf062

202503Revised_Figure_S2_wraf062

202503Revised_Figure_S3_wraf062

202503Revised_Figure_S4_wraf062

202503Revised_Figure_S5_wraf062

202503Revised_Figure_S6_wraf062

Table_S1_wraf062

Table_S2_wraf062

Table_S3_wraf062

20250329_Supplementary_Information_wraf062

## Data Availability

All data needed to evaluate the conclusions in the paper are presented in the paper and/or the Supplementary Information.
