# Supplementary figures and images for: Millimeter-scale niche differentiation of N-cycling microorganisms across the soil-water interface has implications for N2O emissions from wetlands
Source: ISME J. 2025 May 3;19(1):wraf062. doi: 10.1093/ismejo/wraf062 (PMC12270535; doi:10.1093/ismejo/wraf062)

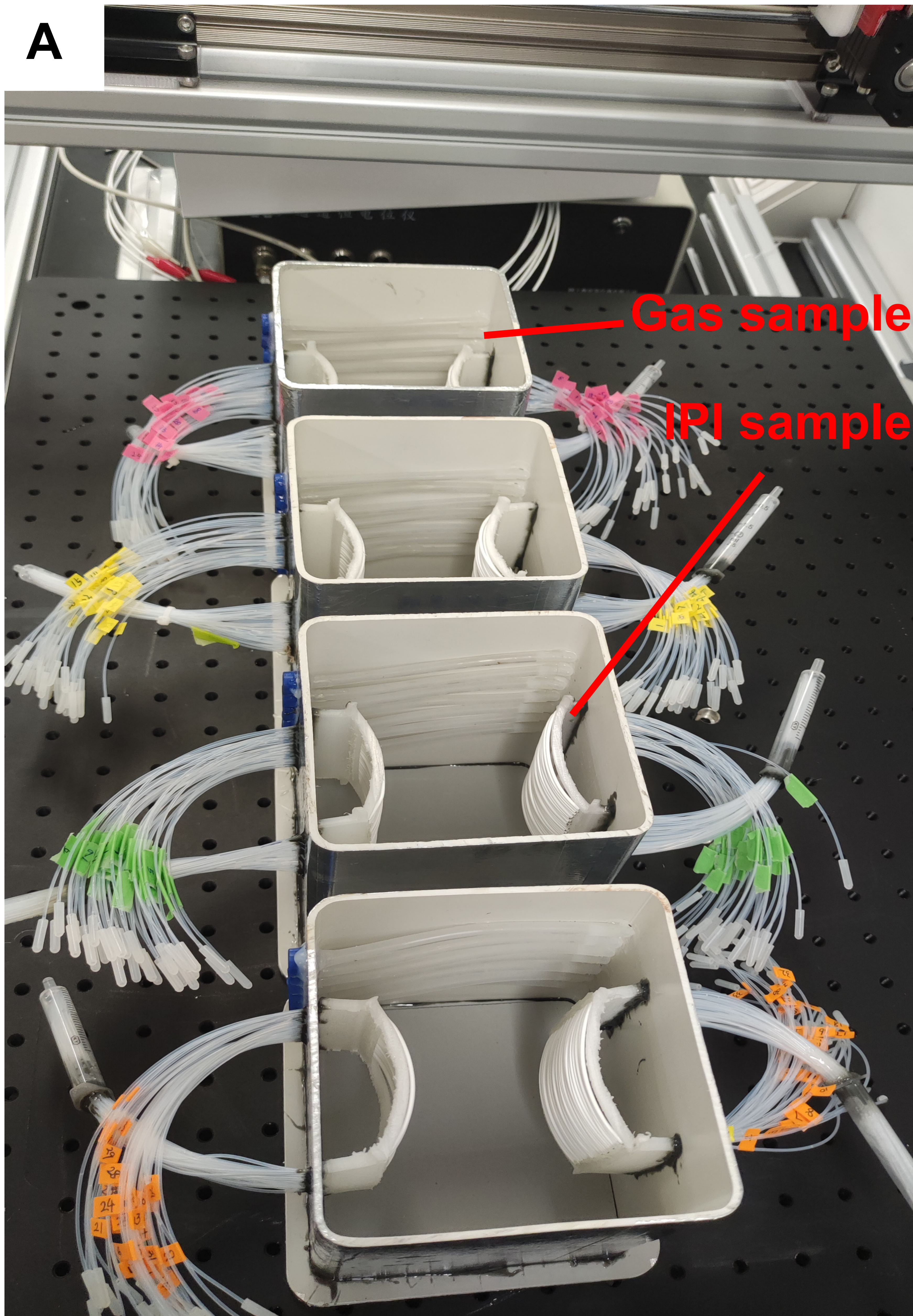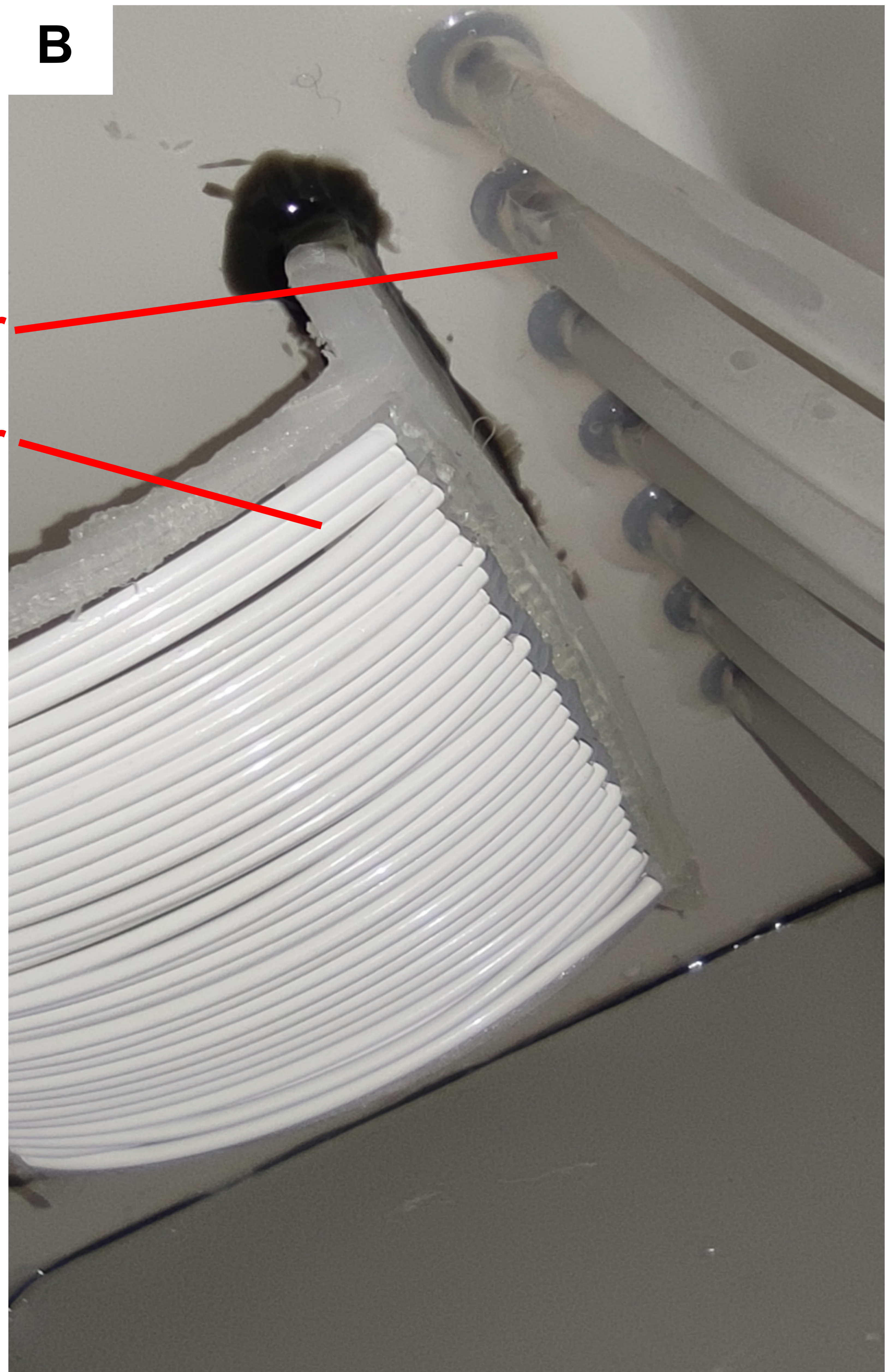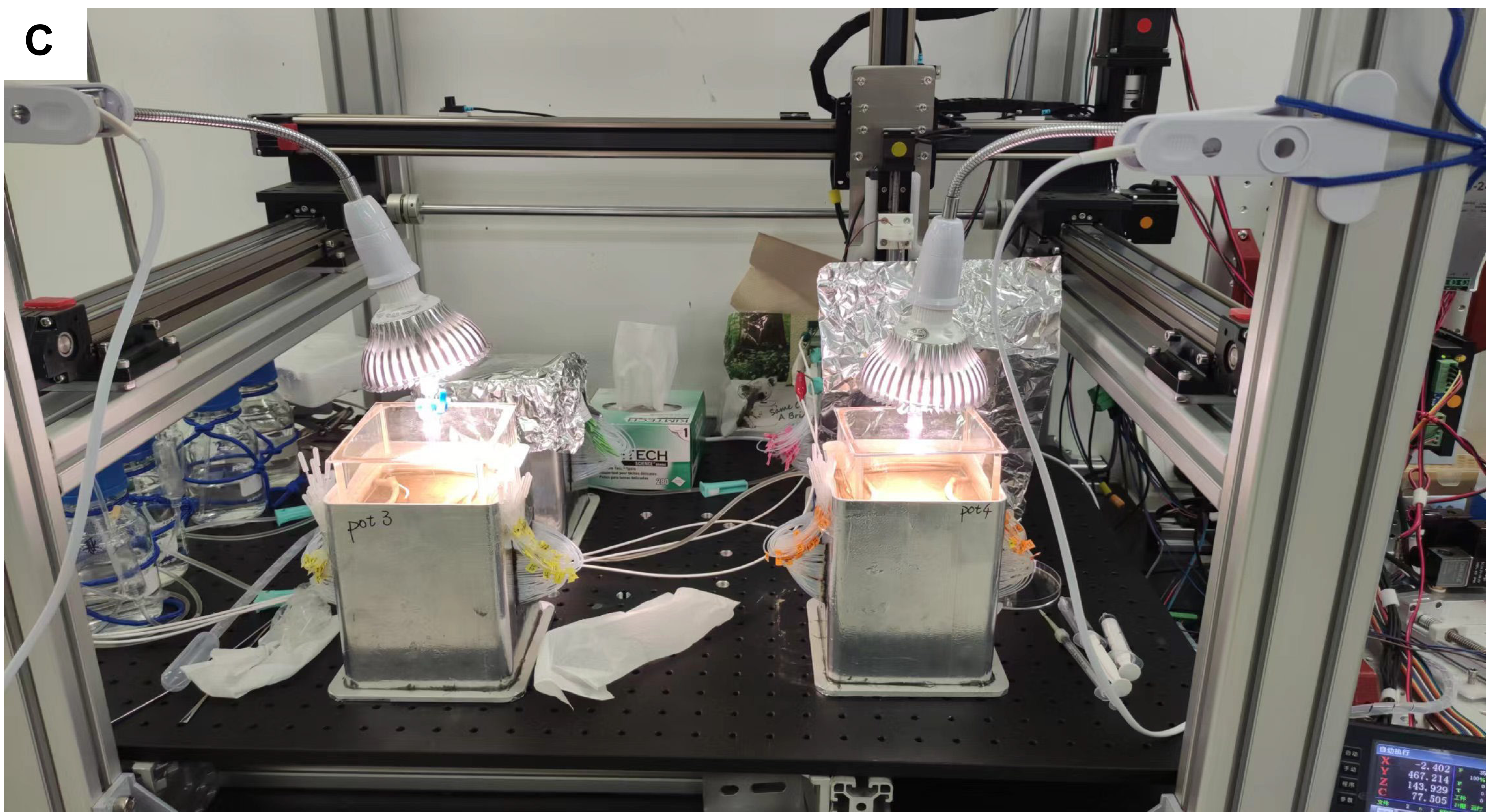

Supplement: 202503Revised_Figure_S1_wraf062 [file 202503revised_figure_s1_wraf062.pdf]

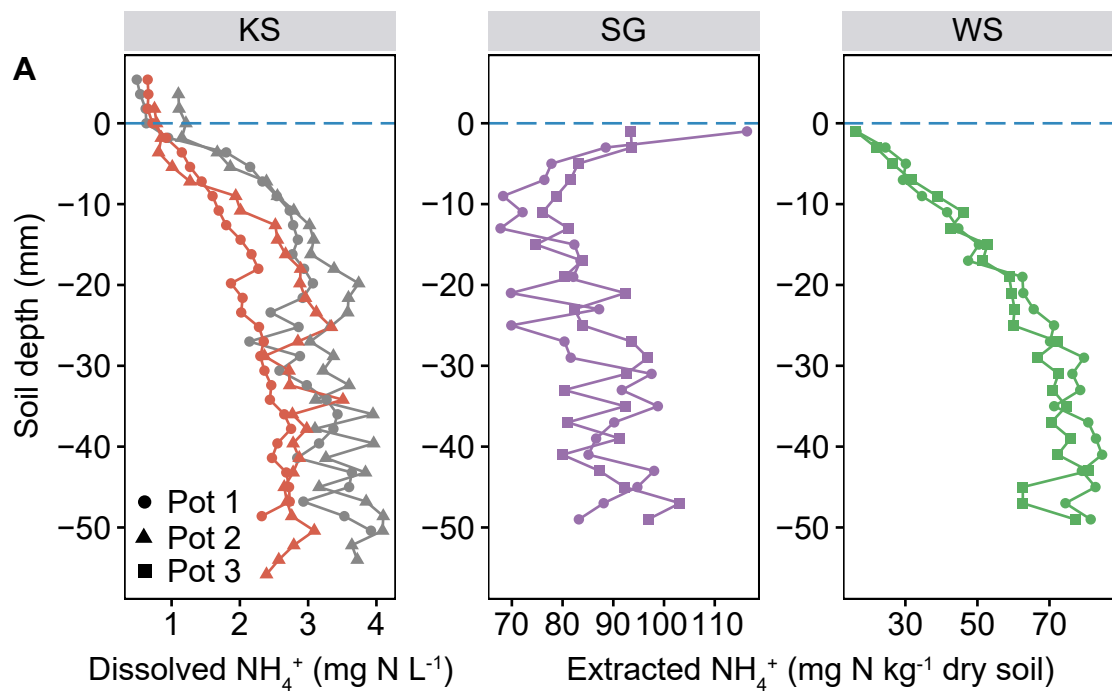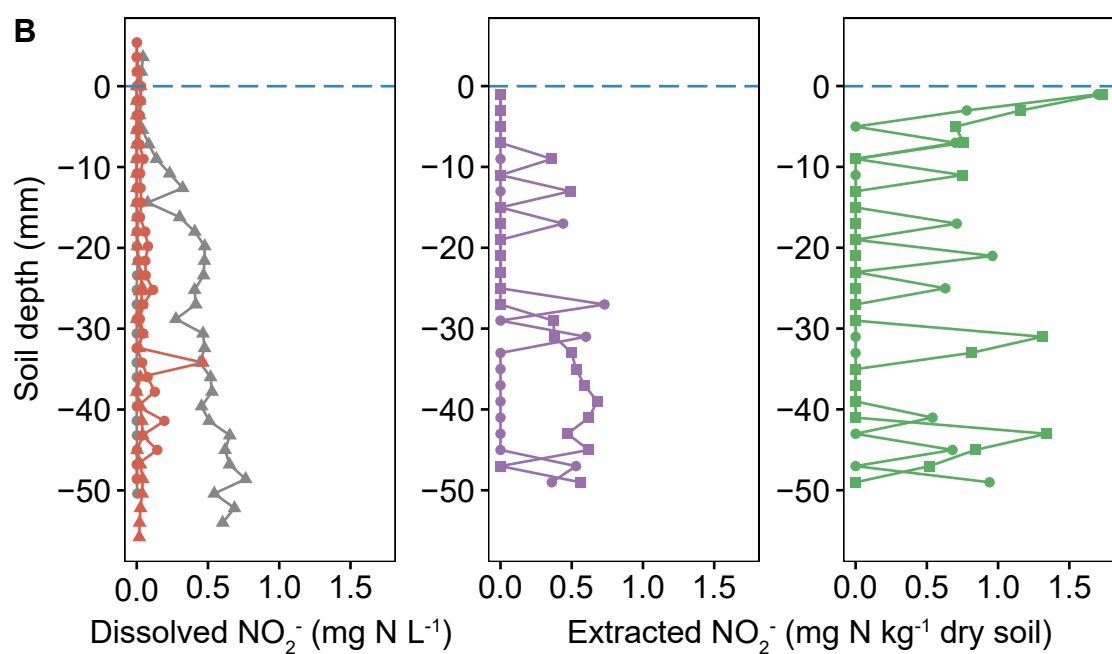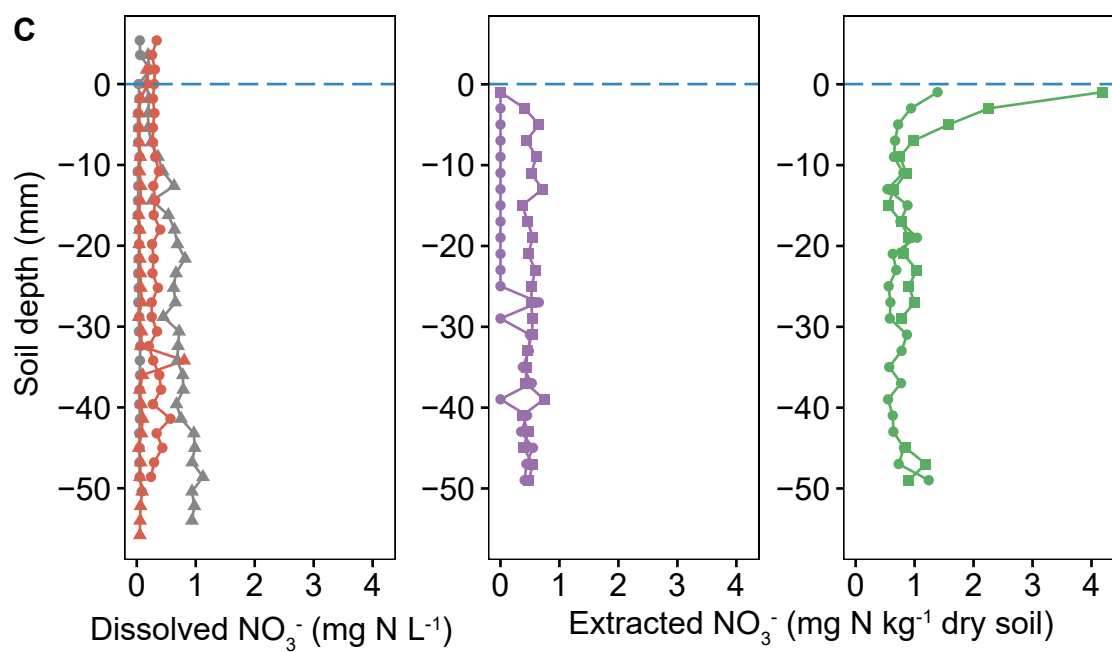

Supplement: 202503Revised_Figure_S2_wraf062 [file 202503revised_figure_s2_wraf062.pdf]

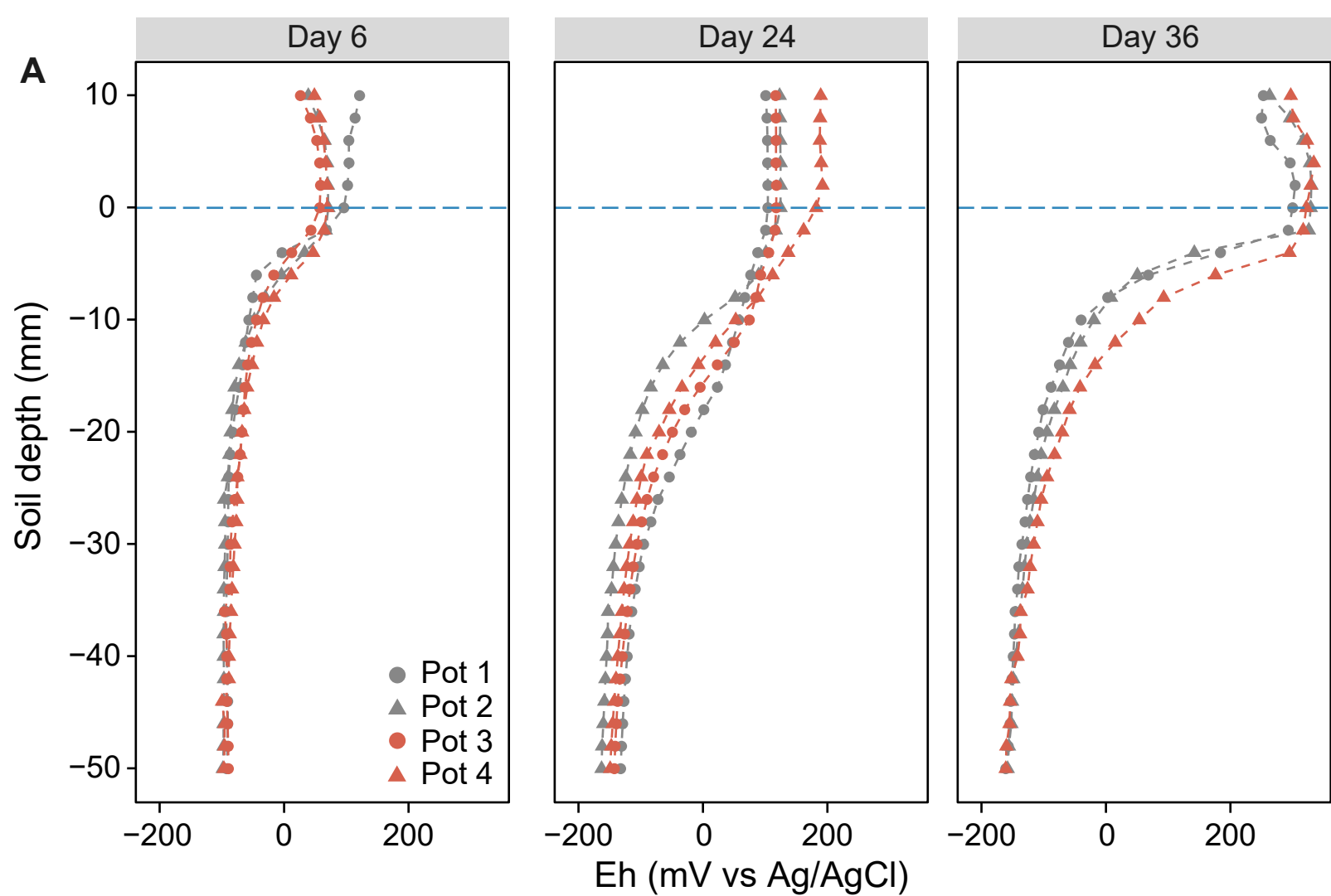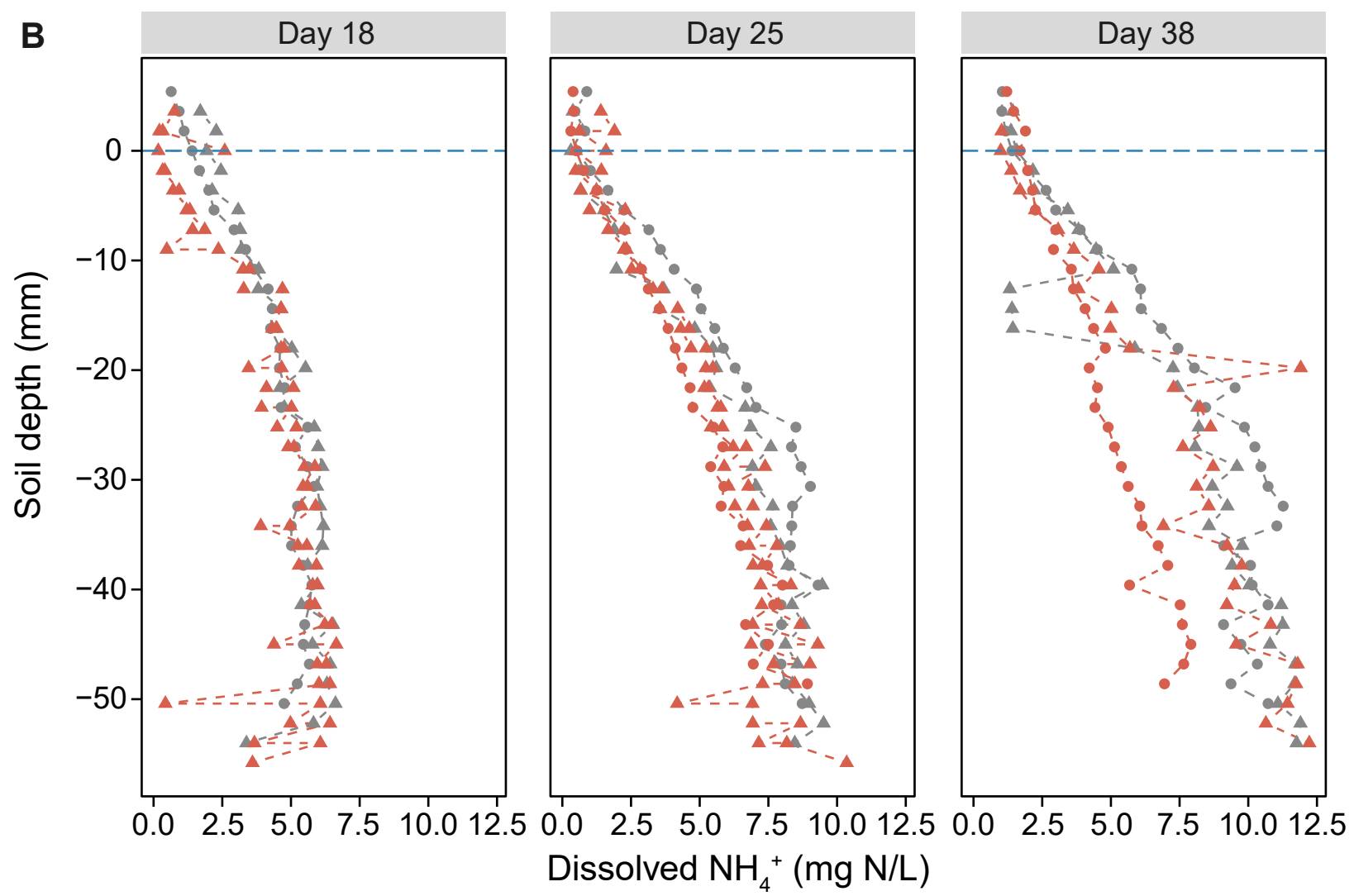

Supplement: 202503Revised_Figure_S3_wraf062 [file 202503revised_figure_s3_wraf062.pdf]

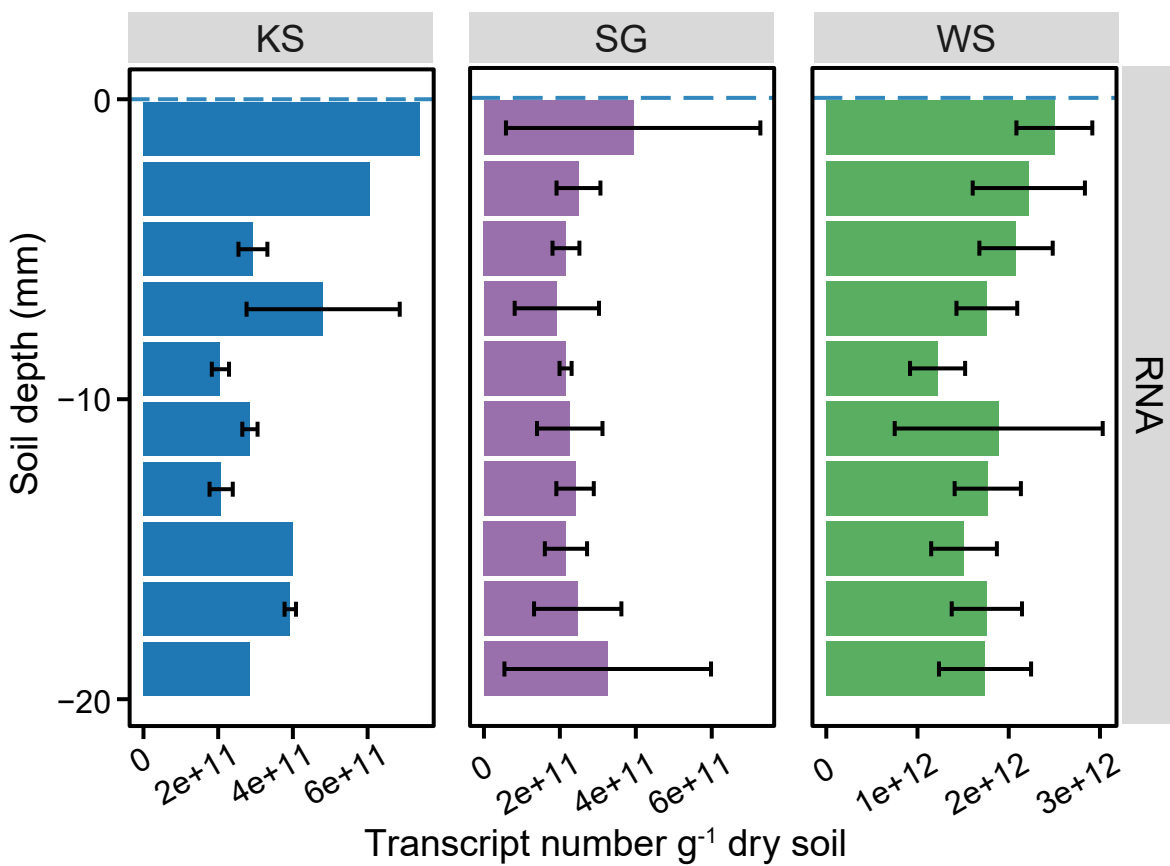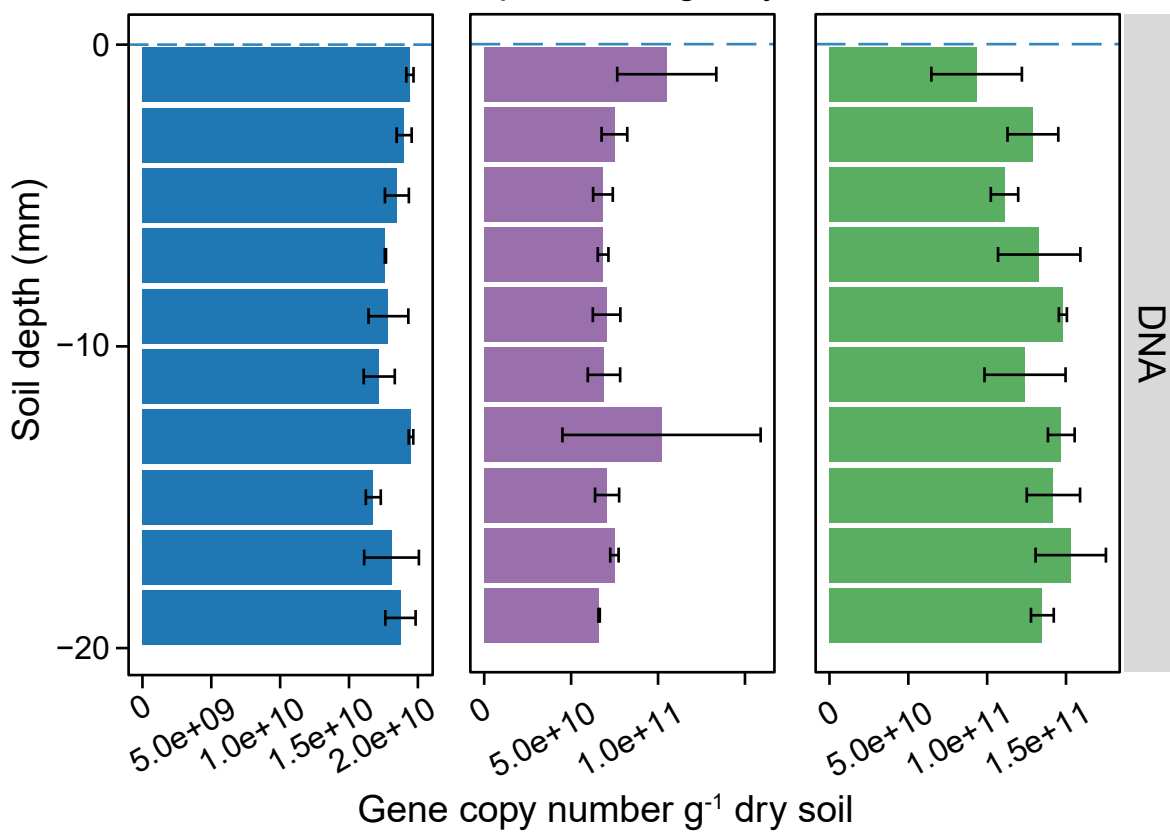

Supplement: 202503Revised_Figure_S4_wraf062 [file 202503revised_figure_s4_wraf062.pdf]

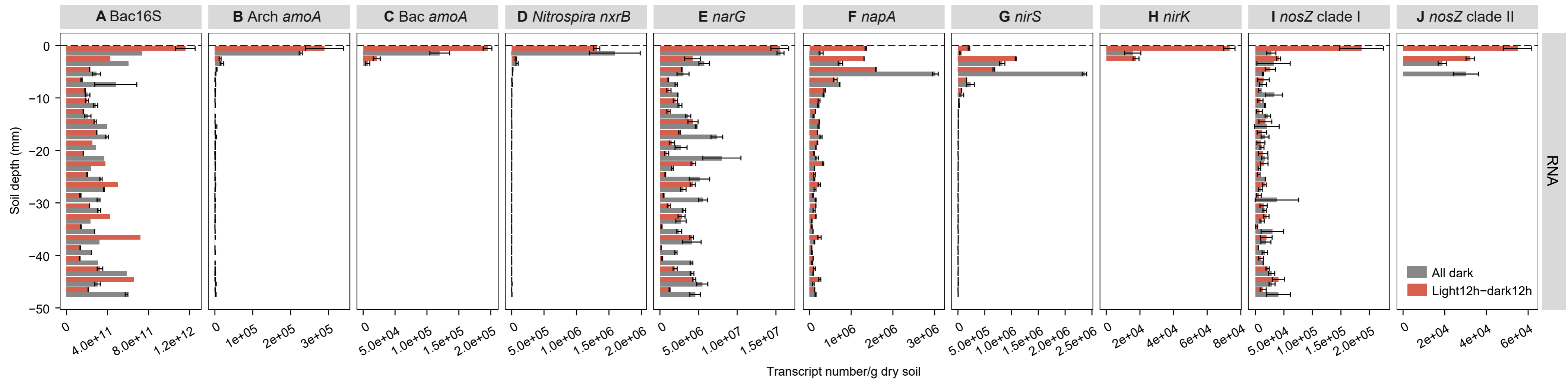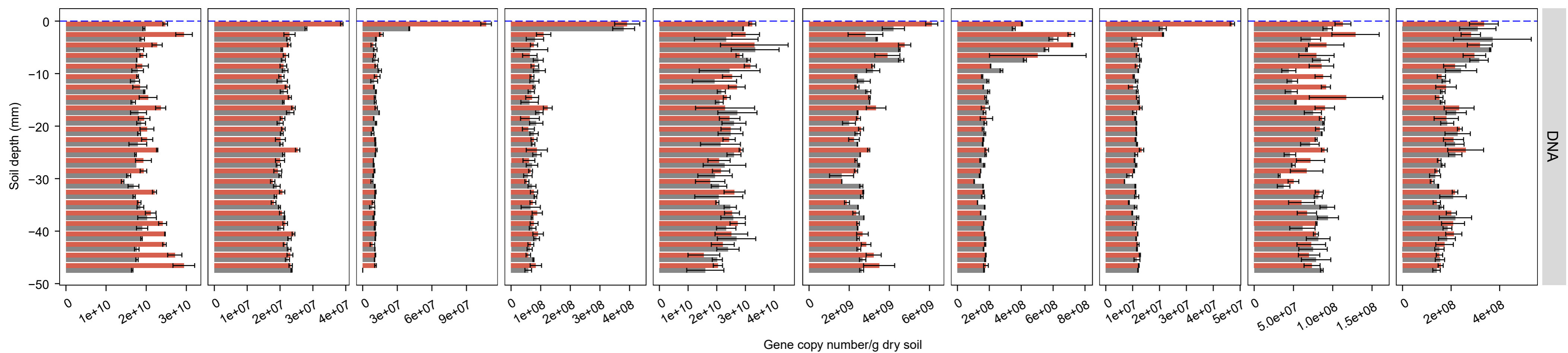

Supplement: 202503Revised_Figure_S5_wraf062 [file 202503revised_figure_s5_wraf062.pdf]

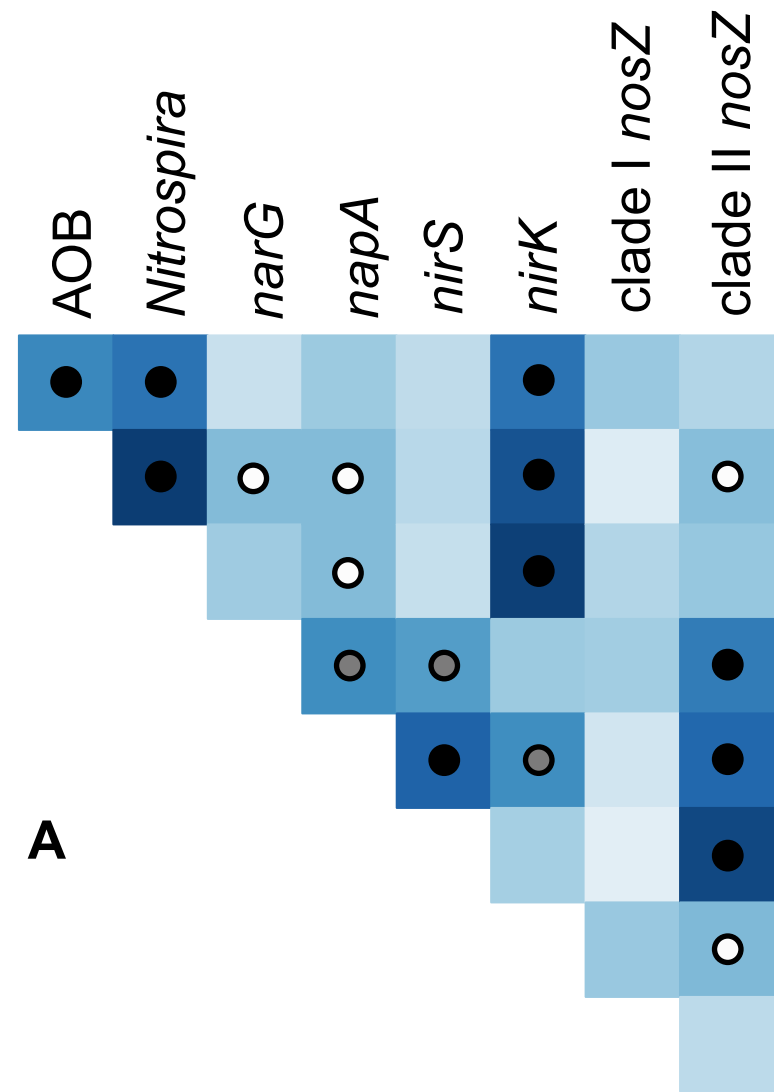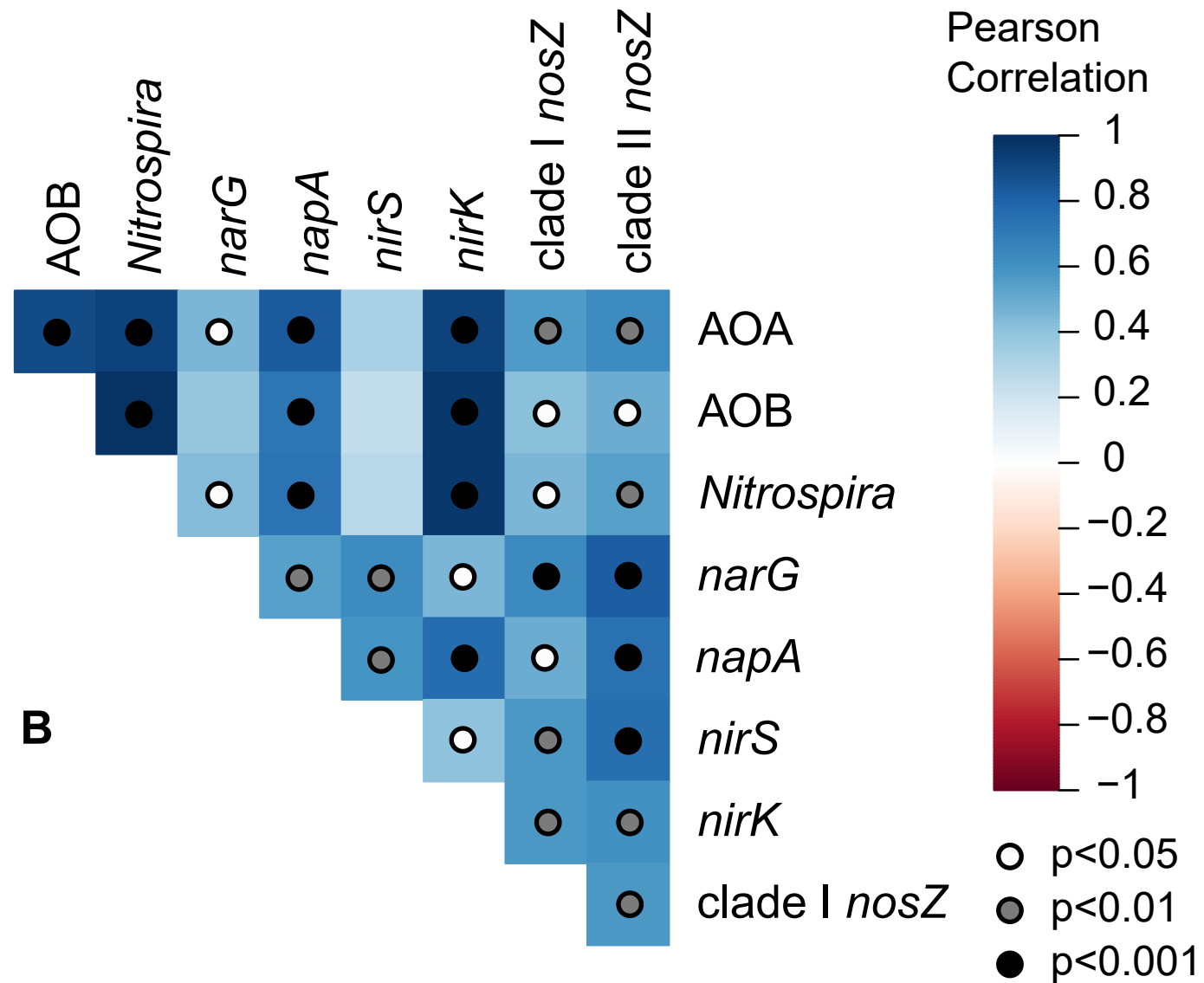

Supplement: 202503Revised_Figure_S6_wraf062 [file 202503revised_figure_s6_wraf062.pdf]
