# Supplementary material for: Millimeter-scale niche differentiation of N-cycling microorganisms across the soil-water interface has implications for N2O emissions from wetlands
Source: ISME J. 2025 May 3;19(1):wraf062. doi: 10.1093/ismejo/wraf062 (PMC12270535; doi:10.1093/ismejo/wraf062)
Supplement: Table_S1_wraf062 [file table_s1_wraf062.docx]

**Table S1.** Basic information and N_2_O emission of the wetland soils sampled across China.

| Sampling site | Province | GPS | Wetland type | pH | DOC^a^  (mg kg^-1^) | DON^a^  (mg kg^-1^) | NH_4_^+^-N  (mg kg^-1^) | NO_2_^-^-N  (mg kg^-1^) | NO_3_^-^-N  (mg kg^-1^) | N_2_O flux in flooding incubation (μg N h^-1^ kg^-1^ dry soil) |
| --- | --- | --- | --- | --- | --- | --- | --- | --- | --- | --- |
| Baoding (BD) | Hebei | 38.92° N, 115.95° E | Reed wetland | 7.91 | 68.2 | 1.5 | 17.0 | 0.3 | 0.3 | ND^b^ |
| Daqing (DQ) | Heilongjiang | 46.58° N, 125.17° E | Rice paddy | 8.34 | 71.6 | 54.9 | 19.5 | 1.0 | 1.4 | ND |
| Ganzhou (GZ) | Jiangxi | 25.29° N, 115.07° E | Rice paddy | 5.18 | 190.6 | 2.8 | 43.0 | ND | ND | ND |
| Kunshan (KS) | Jiangsu | 31.33° N, 120.97° E | Lotus-planting field | 7.69 | 70.2 | ND | 21.7 | ND | ND | 2.22 ± 0.69 |
| Shaoguan (SG) | Guangdong | 25.11° N, 113.64° E | Rice paddy | 5.50 | 136.7 | 2.1 | 20.7 | ND | 0.4 | ND |
| Suzhou (SZ) | Jiangsu | 31.13° N, 120.32° E | Rice paddy | 5.97 | 137.7 | 10.0 | 43.7 | ND | 1.0 | ND |
| Wenshan (WS) | Yunnan | 23.74° N, 105.43° E | Rice paddy | 7.85 | 226.0 | 9.5 | 23.6 | ND | ND | 5.80 ± 0.25 |
| Wuxue (WX) | Hubei | 29.99° N, 115.63° E | Rice paddy | 5.98 | 155.8 | 16.9 | 39.3 | ND | 1.6 | ND |
| Xinxiang (XX) | Henan | 25.28° N, 113.72° E | Rice paddy | 8.24 | 72.2 | 38.6 | 17.3 | ND | 0.2 | ND |
| Taizhou (TZ) | Jiangsu | 31.94° N, 120.18° E | Coastal intertidal zone | 7.63 | 65.5 | ND | 14.6 | ND | ND | ND |

a DOC: dissolved organic carbon; DON: dissolved organic nitrogen.

b ND: not detected
