# Supplementary material for: Millimeter-scale niche differentiation of N-cycling microorganisms across the soil-water interface has implications for N2O emissions from wetlands
Source: ISME J. 2025 May 3;19(1):wraf062. doi: 10.1093/ismejo/wraf062 (PMC12270535; doi:10.1093/ismejo/wraf062)
Supplement: Table_S2_wraf062 [file table_s2_wraf062.docx]

**Table S2.** Site location, wetland type and soil physicochemical characterization of the soils used in the soil mesocosm experiment. Shaoguan (SG) soil see **Table S1**.

| Sampling site | Kunshan (KS) | Wenshan (WS) |
| --- | --- | --- |
| Geological information | 31.4 °N, 120.9 °E | 23.73° N, 105.42° E |
| Sampling time | Feb 2023 | May 2023 |
| Wetland type | Rice paddy | Rice paddy |
| pH | 6.30 | 7.04 |
| TC (g/kg) | 18.1 | 79.2 |
| TN (g/kg) | 1.83 | 6.29 |
| C/N ratio | 9.92 | 12.6 |
| DOC^a^ (mg kg^-1^) | 121 | 158 |
| DON^a^ (mg kg^-1^) | 14.4 | 103 |
| NH_4_^+^-N (mg kg^-1^) | 5.8 | 9.3 |
| NO_2_^-^-N (mg kg^-1^) | ND^b^ | ND |
| NO_3_^-^-N (mg kg^-1^) | ND | ND |

a DOC: dissolved organic carbon; DON: dissolved organic nitrogen.

b ND: not detected
