# Supplementary material for: Millimeter-scale niche differentiation of N-cycling microorganisms across the soil-water interface has implications for N2O emissions from wetlands
Source: ISME J. 2025 May 3;19(1):wraf062. doi: 10.1093/ismejo/wraf062 (PMC12270535; doi:10.1093/ismejo/wraf062)
Supplement: Table_S3_wraf062 [file table_s3_wraf062.docx]

**Table S3.** Information of the primers used in the qPCR assays.

| Primer set | Target gene | N transformation | Sequences (5’-3’) | Reference |
| --- | --- | --- | --- | --- |
| 515F/ 806R | Bacterial 16S rRNA gene V4 | - | GTGCCAGCMGCCGCGGTAA/  GGACTACHVGGGTWTCTAAT | [3] |
| Arch-amoAF/Arch-amoAR | Archaeal *amoA* | NH_3_ monooxygenase | STAATGGTCTGGCTTAGACG/  GCGGCCATCCATCTGTATGT | [4] |
| amoA1F/amoA2R | Bacterial *amoA* | NH_3_ monooxygenase | GGGGTTTCTACTGGTGGT/  CCCCTCKGSAAAGCCTTCTTC | [5] |
| nxrB169f/nxrB638r | *Nitrospira nxrB* | NO_2_^-^ oxidoreductase | TACATGTGGTGGAACA/  CGGTTCTGGTCRATCA | [6] |
| narGF/narGR | *narG* | membrane-bound NO_3_^-^ reductase | TCGCCSATYCCGGCSATGTC/  GAGTTGTACCAGTCRGCSGAYTCSG | [7] |
| V17m/napA4r | *napA* | periplasmic NO_3_^-^ reductase | TGGACVATGGGYTTYAAYC/  ACYTCRCGHGCVGTRCCRCA | [7] |
| nirSC1F/nirSC1R | *nirS* | cytochrome cd1-containing NO_2_^-^ reductase | ATCGTCAACGTCaargaracvgg/  TTCGGGTGCGTCttsabgaasag | [8] |
| nirKC1F/nirKC1R | *nirK* | copper-containing NO_2_^-^ reductase | ATGGCGCCATCatggtnytncc/  TCGAAGGCCTCGatnarrttrtg | [8] |
| nosZ1F/nosZ1R | clade I *nosZ* | N_2_O reductase | WCSYTGTTCMTCGACAGCCAG/  ATGTCGATCARCTGVKCRTTYTC | [9] |
| nosZ-II-F/nosZ-II-R | clade II *nosZ* | N_2_O reductase | CTIGGICCIYTKCAYAC/  GCIGARCARAAITCBGTRC | [10] |
