# Supplementary material for: Millimeter-scale niche differentiation of N-cycling microorganisms across the soil-water interface has implications for N2O emissions from wetlands
Source: ISME J. 2025 May 3;19(1):wraf062. doi: 10.1093/ismejo/wraf062 (PMC12270535; doi:10.1093/ismejo/wraf062)
Supplement: 20250329_Supplementary_Information_wraf062 [file 20250329_supplementary_information_wraf062.pdf]

Supplementary information

Title: Millimeter-scale niche differentiation of N-cycling microorganisms across the soil-water interface has implications for N_2O emissions from wetlands

Running title: N cycling across soil-water interface

Yu-Jia Cai[1,2], Hong-Yang Zhang[1], Xiao-Ran Hu[1], Yu-Chen Yang[1], Christina Hazard[3], Graeme W. Nicol[3], Ji-Zheng He[4,5], Ju-Pei Shen[4], Zi-Yang He[5], Lu Zhang[1], Jing-Hui Zhang[1], Hao Liu[1], Sha Zhang[1], Zheng Chen[1]*

[1] Department of Health and Environmental Sciences, Xi'an Jiaotong-Liverpool University, 111 Ren'ai Road, Suzhou, Jiangsu Province, 215123, China

[2] Department of Geography and Planning, School of Environmental Sciences, University of Liverpool, Brownlow Hill, Liverpool, L697ZX, United Kingdom

[3] Université Claude Bernard Lyon 1, CNRS, INRAE, VetAgro Sup, Laboratoire d'Ecologie Microbienne, Villeurbanne, 69622, France

[4] Key Laboratory of Humid Subtropical Eco-geographical Process of Ministry of Education, School of Geographical Sciences/School of Carbon Neutrality Future Technology, Fujian Normal University, Fuzhou, Fujian Province, 350007, China

[5] School of Agriculture, Food and Ecosystem Sciences, Faculty of Science, The University of Melbourne, Parkville, VIC, 3010, Australia

*Corresponding author: Zheng Chen; Department of Health and Environmental Sciences, School of Science, Xi'an Jiaotong-Liverpool University, 111 Ren'ai Road, Suzhou, Jiangsu Province, 215123, China. Email: ebiogeochem@outlook.com, Zheng.Chen@xjtlu.edu.cn

Configuration and assemble of the integrated porewater injection (IPI) sampler

Based on the microdialysis concept, the IPI sampler [1, 2] was used to sample *in situ* the soil porewater. Briefly, the sampler was a 3D-printed skeleton holder (62 mm outer width × 86 mm height) with 33 nanomembrane sampling tubes (polytetrafluoroethylene, PTEF) installed to sample the 10 mm water layer and 50 mm soil layer (Fig S1a, b). The size for the nanomembrane tube is 1.0 mm inner diameter × 1.7 mm outer diameter × 54 mm length. The sampling resolution for the soil porewater is therefore 1.8 mm. The sampler was filled with oxygen-free ultrapure water (18.2 MΩ) for the duration of the experiment. When sampling soil porewater, preloaded water was removed and replaced with deoxygenated ultrapure water. After 6 h when equilibrium was reached (determined via preliminary tests, data not shown), approximately 200 µL of soil porewater was sampled for analyses of N species.

Table legends

Table S1. Basic information and N_2O emission of the wetland soils sampled across China.

Table S2. Site location, wetland type and soil physicochemical characterization of the soils used in the soil mesocosm experiment. Shaoguan (SG) soil see **Table S1**.

Table S3. Information of the primers used in the qPCR assays.

Figure legends

Figure S1. Diagram of the experimental system. (A) and (B) Pot container implanted with the Integrated Porewater Injection (IPI) sampler and gas sampling tubes. (C) N_2O flux measurement taken during a period of light illumination.

Figure S2. Depth profiles of (A) NH_4^+, (B) NO_2^- and (C) NO_3^- across the SWI at the end of the mesocosm experiment. In KS soil, gray dots represent the all-dark group while orange represent the light-dark group. The inorganic N of KS soil was retrieved with the IPI sampler. Two mesocosms replicates for SG and WS soils were randomly selected for the extraction of inorganic N analysis.

Figure S3. Temporal change of (A) Eh and (B) dissolved NH_4^+ across the SWI of KS soil. The four mesocosms can be considered as replicates until they were divided and subjected to light-dark cycling on day 39. Mesocosm information is consistent with the KS soil in Figure. S2.

Figure S4. Depth profiles of the bacterial 16S rRNA gene and transcript. Error bar represents the standard error of the mean of technical replicates of KS soil microcosm and biological replicates (n=3) of SG and WS mesoocosms. No biological replicates are available for KS soil.

Figure S5. Depth profiles of abundance and transcript number of (A) bacterial 16S rRNA gene and (B-J) N-cycling microorganisms in KS soil. Gray column represents the all-dark

group, while orange represents the light-dark group. E Error bar represents the standard error of the mean of technical replicates. No biological replicates are available for KS soil.

Figure S6. Correlations between the abundance of N-cycling microorganisms in the A) all-dark and B) light-dark group of KS soil.

References

1. Yuan ZF, Gustave W, Bridge J, Liang Y, Sekar R, Boyle J, et al. Tracing the dynamic changes of element profiles by novel soil porewater samplers with ultralow disturbance to soil-water interface. *Environ Sci Technol* 2019;53:5124-32. https://doi.org/10.1021/acs.est.8b05390

2. Zhang S, Yuan Z, Cai Y, Liu H, Liu ZY, Chen Z. Dissolved solute sampling across an oxic-anoxic soil-water interface using microdialysis profilers. *J Vis Exp* 2023;193:e64358. https://doi.org/10.3791/64358-v

3. Caporaso JG, Lauber CL, Walters WA, Berg-Lyons D, Lozupone CA, Turnbaugh PJ, et al. Global patterns of 16S rRNA diversity at a depth of millions of sequences per sample. *Proc Natl Acad Sci USA* 2011;108:4516-22. https://doi.org/10.1073/pnas.1000080107

4. Francis CA, Roberts K, Beman JM, Santoro AE, Oakley BB. Ubiquity and diversity of ammonia-oxidizing archaea in water columns and sediments of the ocean. *Proc Natl Acad Sci USA* 2005;102:14683-8. https://doi.org/10.1073/pnas.050662510

5. Rotthauwe JH, Witzel KP, Liesack W. The ammonia monooxygenase structural gene *amoA* as a functional marker: molecular fine-scale analysis of natural ammonia-oxidizing populations. *Appl Environ Microbiol* 1997;63:4704-12. https://doi.org/10.1128/aem.63.12.4704-4712.1997

6. Pester M, Maixner F, Berry D, Rattei T, Koch H, Lücker S, et al. *NxrB* encoding the beta subunit of nitrite oxidoreductase as functional and phylogenetic marker for nitrite-oxidizing *Nitrospira*. *Environ Microbiol* 2014;16:3055-71. https://doi.org/10.1111/1462-2920.12300

7. Bru D, Sarr A, Philippot L. Relative abundances of proteobacterial membrane-bound and periplasmic nitrate reductases in selected environments. *Appl Environ Microbiol* 2007;73:5971-4. https://doi.org/10.1128/AEM.00643-07

8. Wei W, Isobe K, Nishizawa T, Zhu L, Shiratori Y, Ohte N, et al. Higher diversity and abundance of denitrifying microorganisms in environments than considered previously. *ISME J* 2015;9:1954-65. https://doi.org/10.1038/ismej.2015.9

9. Henry S, Bru D, Stres B, Hallet S, Philippot L. Quantitative detection of the *nosZ* gene, encoding nitrous oxide reductase, and comparison of the abundances of 16S rRNA, *narG*, *nirK*, and *nosZ* genes in soils. *Appl Environ Microbiol* 2006;72:5181-9. https://doi.org/10.1128/AEM.00231-06

10. Jones CM, Graf DRH, Bru D, Philippot L, Hallin S. The unaccounted yet abundant nitrous oxide-reducing microbial community: a potential nitrous oxide sink. *ISME J* 2013;7:417-26. https://doi.org/10.1038/ismej.2012.125
